# Deep brain stimulation for post-stroke rehabilitation in Pakistan

**DOI:** 10.1097/MS9.0000000000002511

**Published:** 2024-08-30

**Authors:** Muhammad Idrees, Muskan A. Taimuri, Ayesha Azhar, Najam A. Khan, Umme Kalsoom, Aiman Sajid, Aymar Akilimali

**Affiliations:** a University of Health Sciences, Lahore; b Department of Internal Medicine, Dow University of Health Sciences; c Sir Syed College of Medical Sciences, Karachi; d Ayub Medical College, Abbottabad, Pakistan; e Department of Research, Medical Research Circle, Goma, DR Congo

**Keywords:** deep brain stimulation, Pakistan, rehabilitation, stroke

## Abstract

Stroke has a high prevalence in Pakistan, at an alarming rate of 250 per 100 000 people. Although various treatment options are available, they are not ideal for Pakistan due to their high cost, restricted availability, and time sensitivity. In 1997, the FDA-approved deep brain stimulation (DBS) for Parkinson’s disease and it was first performed in Pakistan in 2014. DBS has also proved effective for restoring post-stroke mobility, according to a trial from August 2023. DBS has the potential to revolutionize post-stroke rehabilitation in Pakistan; however, further research is required into its effectiveness and its limitations must be addressed first.

## Introduction

Highlights

- Stroke is the second leading cause of death, with 5.5 million deaths due to stroke reported in 2020.
- Lower middle-income countries (LMICs) have a higher incidence of stroke, accounting for 86% of the deaths.
- Stroke has a high prevalence in Pakistan, at an alarming rate of 250 per 100 000 people.
- Although various treatment options are available, they are not ideal for Pakistan due to their high cost, restricted availability, and time sensitivity.
- In 1996, the FDA-approved deep brain stimulation (DBS) for treating essential tremor in Parkinson’s disease. DBS has also proved effective for restoring post-stroke mobility, according to a trial from August 2023.
- DBS was first performed in Pakistan at the Lahore General Hospital in 2014 to treat Parkinson’s disease.
- DBS holds great promise for transforming post-stroke rehabilitation in Pakistan; yet, additional investigation is necessary to determine its efficacy, and its constraints must be addressed prior to any progress being made.

Stroke is a cerebrovascular event caused by vascular occlusion or leakage, leading to complex manifestations with respective focal areas of the brain^1^. The most common type, ischaemic stroke, results from a thromboembolic event and accounts for 87% of cases. The other type, haemorrhagic stroke, is caused by the rupture of a blood vessel and constitutes 13% of cases^2^. Compromised blood flow in either case causes necrosis of neuronal cells, which aggravates a cascade of various molecular mechanisms at the cellular level. Key microscopic mechanisms contributing to stroke-related disability include porous cellular membranes, energy crises, acidic pH, high calcium-induced free radical injuries, and neuroinflammation^1^.

Stroke is the second leading cause of death worldwide, with approximately 5.5 million stroke-related fatalities reported in 2020^2^ and 15 million individuals affected by stroke in 2021^1^. Low- and middle-income countries (LMICs), particularly in South Asia, have a higher prevalence of stroke than Europe or the United States^3^. In 2022, the World Stroke Organization (WSO) estimated a 70% increase in worldwide cases of incident stroke, from 1990 to 2019, with a 43% increase in mortality and a staggering 143% increase in daily-adjusted life years (DALYs)^4^. Out of these, 86% deaths and 89% DALYs were contributed by low-income countries and low- and middle-income countries.

In Pakistan, the first population-based study to assess the prevalence of stroke conducted in 2006 reported a prevalence rate of 4.8% among ethnic Pashtuns in Karachi^5^. According to a 2018 study, the incidence of stroke in Pakistan was 95 per 100 000 people between 2000 and 2016, which surged to an alarming 250 per 100 000 people in 2018^1^. Additionally, while stroke is generally associated with older age in developed countries, multiple studies have reported a significant prevalence of stroke among younger individuals in Pakistan. For instance, a 2020 study reported that over 66% of patients with stroke in Khyber Pakhtunkhwa, Pakistan were younger than 50 years^5^. In addition to non-modifiable risk factors such as age, race, and sex, various modifiable factors such as hypertension, diabetes, dyslipidemia, obesity, and ischaemic heart disease are also associated with an increased risk of stroke in Pakistan^1^.

In this review, we explore the current treatment options available for stroke, emphasizing the novel deep brain stimulation (DBS) procedure as a promising approach for post-stroke rehabilitation in LMICs like Pakistan where stroke prevalence is strikingly high.

## Challenges and treatment modalities for stroke in Pakistan

Various therapeutic approaches, including thrombolysis, anti-platelet agents, and fibrinogen-lowering drugs are employed for acute stroke care, while physical, speech, and occupational therapies are utilized for rehabilitation of post-stroke complications^2^. Some of the most common post-stroke complications include pain, paralysis, bowel and bladder atony, cognitive impairment, depression, and permanent disability^6^. Complications prevalent in Pakistan include falls post-stroke (38.8%), hemiplegic shoulder (27.4%), mild dementia (14.8%), dysphagia (13.7%), urinary tract infections (6%), severe neurological pain (4%), pressure ulcers (2.2%) and deep venous thrombosis (1.1%)^6^.

Neuroprotective agents, including GABA agonists (clomethiazole), sodium channel blockers (mexiletine), and antioxidants (NXY-059) as well as stem cell therapy for neuronal repair, can be employed to prevent disability^1^. In Pakistan, various treatment modalities have been introduced, including thrombolysis, endovascular surgery for ischaemic stroke, and endovascular coiling or clipping for haemorrhagic stroke. However, the high cost significantly limits the availability of these options. Endovascular treatments, such as coiling for haemorrhagic stroke, have demonstrated a 91.7% success rate in Pakistan but are constrained by a shortage of tertiary care facilities in the country and adequately trained medical professionals^7^. Intravenous thrombolysis with alteplase (tissue plasminogen activator, tPA) has shown considerable efficacy in the management of acute ischaemic stroke but its high cost, US $2250 per injection, renders it impractical for Pakistan, an LMIC with a per capita income of only US$450^8^.

These post-stroke complications are often poorly managed in Pakistan due to scarcity of neurology experts, poorly established neuro-rehabilitation facilities, and cultural beliefs that lead to delays in accessing care for stroke-related complications^9,10^. Pakistan’s healthcare infrastructure is significantly underfunded, with merely1.4% of the total Gross Domestic Product (GDP) allocated to healthcare in 2022^11^. The country faces a notable scarcity of neurologists, with approximately 200 neurologists for 200 million people as of 2020^9^, and only 80 trained rehabilitation medicine physicians serving nationwide^10^. Although the Pakistan Stroke Society has been working to improve stroke facilities in Pakistan, the country still hosts only 10 specialized stroke centres^10^.

## DBS: mechanism, clinical applications, and efficacy

The Food and Drug Administration (FDA) approved DBS of the ventral intermediate nucleus (VIM) of the thalamus in 1997 for essential tremor and severe tremor in Parkinson’s disease^12^. DBS is a pioneering medical procedure used for the treatment of various neuropsychiatric conditions, including movement disorders associated with Parkinsonian disease, essential tremors, dystonias, obsessive-compulsive disorder (OCD), and epilepsy. This neurosurgical procedure involves the implantation of single or multiple stimulating electrodes, known as leads, into the brain parenchyma, which are connected to an implantable pulse generator (IPG), similar to cardiac pacemakers^13^. This IPG, implanted under the skin of the upper chest delivers finely adjusted electrical impulses to specific brain structures^13^. The stimulation can be rapidly switched on and off, ranging from split-second intervals to several seconds. However, it is most commonly delivered continuously around the clock^14^.

Deep brain stimulation is categorized based on the particular brain structure it aims to target, such as the thalamus for the management of essential tremors, globus pallidus or subthalamic nuclei for Parkinson’s disease, and the anterior limb of the internal capsule for OCD^14^. The electrical stimulation to the brain modulates abnormal neural activity patterns that cause tremors, thereby reducing or eliminating symptoms. The mechanism of DBS most likely involves the modulation of neuronal cells and induction of action potentials in nearby axons^13^.

Since its inception, DBS has been used in other countries to treat Parkinson’s disease and was approved for the treatment of dystonia in 2002^13^. According to a clinical trial involving patients with PD, unilateral subthalamic nuclei DBS not only significantly improved depression but also succeeded in enhancing sleep patterns and the overall quality of life^15^. DBS is a reversible and adjustable treatment, which means that the level of stimulation can be fine-tuned to optimize symptom control while minimizing side effects^14^. Life-threatening adverse events, such as brain haemorrhage, are uncommon, occurring in less than 1–2% of the patient population. Less severe, typically reversible events such as wound infections and stimulation-related side effects occur in up to 9% of patients^14^. DBS has emerged as a transformative medical technique, effectively supplanting previously used lesional procedures for the treatment of neurologic conditions^16^.

## DBS for post-stroke movement disorders

DBS has also garnered attention for its potential role in managing stroke-related disorders. Preclinical studies in rodents have demonstrated that cerebellar DBS is an effective treatment modality for post-stroke movement disorders, concurrently promoting synaptic plasticity and neurogenesis in the perilesional cortex^17^. Post-stroke movement disorders are categorized as disorders affecting the basal ganglia, which comprise structures including the striatum (caudate and putamen), globus pallidus, subthalamic nucleus, and substantia nigra. While the thalamus isn’t part of the basal ganglia, it acts as a vital relay station, connecting basal ganglia nuclei to descending motor pathways. In post-stroke movement disorders, ischaemic damage and cell death within the basal ganglia’s direct and indirect pathways lead to abnormal movement regulation. Exogenous electrical stimulation into these circuits via DBS results in the modulation of network activity, which generally leads to the promotion of intended movements or suppression of unintended movements^17^. A 2020 case study by University of Liverpool researchers explored the efficacy of DBS for two patients experiencing post-thalamic stroke movement disorders, specifically dystonia and tremor^18^. Subsequent to DBS intervention targeting either the VIM thalamic nucleus or the globus pallidum internal (GPi) within the basal ganglia, both patients exhibited marked improvement, characterized by a reduction in tremor intensity and an elevation in quality of life^18^.

## The electrical stimulation of the dentate nucleus for upper Extremity Hemiparesis Due to Ischaemic Stroke (EDEN) study

In August 2023, the results of a Phase 1 FDA-approved clinical trial investigating the safety and efficacy of DBS for the treatment of post-stroke motor deficits were released. This trial, known as the Electrical Stimulation of the Dentate Nucleus for Upper Extremity Hemiparesis Due to Ischaemic Stroke (EDEN) study was an open-label, non-randomized phase I trial that recruited twelve patients with persistent (1–3 years) moderate to severe degree of motor disability of the upper limb following an episode of stroke in the middle cerebral artery^19,20^. DBS was applied to the cerebellar dentate nucleus combined with renewed physical rehabilitation to promote functional reorganization of ipsilesional cortex Patients were given three months of rehabilitation after the DBS surgery. The results showed that participants demonstrated a seven-point median improvement on the Upper-Extremity Fugl-Meyer Assessment. Regardless of the time elapsed since the stroke; all individuals who enroled with partial preservation of distal motor function surpassed the minimal clinically important difference, with a median improvement of 15 Upper-Extremity Fugl-Meyer Assessment points. Significant improvements were noted in participants, even those who enroled as late as 3 years following their initial episode.

These preliminary findings indicate potential for enhancing functional recovery after cerebral ischaemia, even in the later stages of disability. Throughout the trial, a total of 168 participant-months of DBS implant experience and 72 months of DN stimulation experience were accumulated. There were no instances of study-related adverse events or device failures reported^19^. Of the recorded adverse events during the trial, totalling 51, 21 were deemed associated with study participation^19^. Pain and nausea were the most common adverse events reported by participants^19^. However, the study excluded patients with uncontrolled hypertension and severe cardiovascular disorders, thus lacking data on patients with these co morbidities. It also failed to provide a comparative analysis of DBS with other non-surgical rehabilitation measures, such as physiotherapy, psychotherapy, and speech therapy, which could have been useful in comparing the overall costs and benefits.

It should be noted, however, that early findings, even in preliminary stages, can lay the foundation for ground-breaking advancements in healthcare. One prime example of this is the development of Tirzepatide, a dual GIP/GLP-1 receptor agonist for the treatment of type 2 diabetes. Initial phase 2 trials yielded promising results, demonstrating Tirzepatide’s potential to significantly reduce haemoglobin A1C levels and promote weight loss. These early successes prompted further research and the drug has shown remarkable efficacy in subsequent phase 3 trials, becoming a highly effective therapy for managing diabetes and obesity^21^. Consequently, although further research is required into the safety and efficacy of DBS, preliminary studies suggest DBS could be a game-changer in the management of stroke and the treatment of stroke-related motor disorders worldwide.

## Clinical efficacy of DBS for stroke

The efficacy of DBS in resolving post-stroke pain largely depends on the area of stimulation. Hamani and colleagues found that simultaneous stimulation of both somatosensory (VPL/VPM thalami) and periaqueductal grey hampered pain more effectively than that of the ventral striatum and anterior limb of the internal capsule demonstrated in a study by Lempka and colleagues in which pain relief was achieved by only one patient as enumerated in Table 1 ^22,29^. Interestingly, stimulation of the nucleus accumbens (part of the ventral striatum) has been found effective in controlling pain^23^. In addition to various targets, DBS’s intensity, voltage, and duration were also found to be additional factors for post-stroke symptoms like pain, dystonia, and paresthesias. Improvement in motor and paraesthesia required 100 Hz intensity for 60 ms but at different voltages 0.6V and 1V respectively as reported by Franzini *et al*.^24^. In contrast to this, stimulation of the ventral intermediate nucleus (VIM)and nucleus ventralis oralis (VO) at 120–185 Hz with 1.7 to 4-volt control tremors while GPi requires 130–190 Hz and 3–6 volts for a similar effect^13^. Dyskinesias secondary to stroke more frequently improves after stimulation at VO/VIM with 25–185Hz but variable parameters like involved pathways and lesion area may change outcomes^13,30^.According to Table 1, we quoted only one study reporting DBS-related complications, supporting the post-op safety profile of DBS. Its application is not only restricted to post-stroke pain but also amplified for dystonias, dyskinesias, and tremors, depending on the focus being stimulated during DBS, as enumerated in Table 1. For instance, targeting globas pallidus pars interna relieved post-stroke dystonias in 5/7 patients as reported by Slow *et al*.^28^. Similarly, 20/28 patients with choreoathetosis and hemiballismus reported improved outcomes after using DBS in another study^27^. Mendonça *et al*.^26^ found recovery of tremors among 82 stroke patients without any postoperative complications of DBS. We discussed these studies in detail as follows.

**Table 1:** Clinical efficacy of DBS for stroke.

Author (year)	Type of study, no. patients included in the study	The post-stroke clinical problem as the rationale behind using DBS	Target outcome	Targets in brain	Clinical advantages of DBS among stroke patients	Complications
Lempka *et al.* (2017)^22^	Double-blind Clinical trial, 10 stroke patients	Pain	More than 50% improvement in pain disability index (PDI)	Ventral striatum and anterior limb of internal capsule	Only one patient achieved the target outcome	Changes in sleep patterns, Headaches, Seizures, wound infections, and altered bowel habits
Mallory *et al.* (2012)^23^	Case report	Pain	Sustained relief till 1 year after DBS	Nucleus accumbens	100% achievement in the primary outcome	No complications reported
Franzini *et al.*(2008)^24^	Case report	Spasticity and paraesthesia	Improved chronic spasticity and paraesthesia	The inferior limb of the internal capsule	Up to 70% improvement was observed	No complication was reported
Franzini *et al.* (2019)^25^	Retrospective case series, 4 stroke patients	Pain and motor disability	Long-term (5 years ) Pain relief and Significant improvement in motor disability	Posterior limb of the internal capsule	3/4 patients achieved long-term pain relief and 2/4 patients improved motor power with a marked reduction in spasticity	No complication was found
Baker *et al.* (2023)^19^	Phase I non-randomized Clinical trial,12 stroke patients	Reduced upper limb motor power	Median improvement in 15 upper-extremity Fugel-Meyer-Assessment points	Cerebellar Dentate Nucleus	All patients achieved 7 points on the designed scale showing marked change in motor power	No perioperative or postoperative short or long-term side effect was observed
Mendonça *et al.* (2018)^26^	Case reports and case series comprising 82 patients	Tremors	Improvement in tremors	The ventral intermediate nucleus (VIM) of the thalamus	Median Improvement in tremor was up to 77.5% among stroke patients	No complications were found
Katayama *et al.* (2006)^27^	Non- randomized clinical study,28 patients	Dyskinesias (Choreoathetosis and Hemiballismus) secondary to stroke	Excellent Control over movement disorders	Motor cortex	20/28 patients successfully achieved control over these movement disorders	No obvious complications were recorded
Slow *et al.* (2015)^28^	Retrospective, observational study,07 patients	Dystonia	Improvement in dystonia-related disability	Globas pallidus pars interna	5/7 patients found to have better outcomes in the form of improved disability	No complications seen

DBS, deep brain stimulation.

## Applications and limitations of DBS in Pakistan

In Pakistan specifically, DBS was first introduced in 2014, when it was employed at Lahore General Hospital for the treatment of Parkinson’s disease^31^. Over the next few years, the DBS program expanded all over the country. The most recent prospective study conducted at the Neurospinal and Cancer Care Institute, Karachi between 2016 and 2020, aimed to assess the efficacy of DBS for patients diagnosed with Parkinson’s disease^32^. With an experimental group consisting of 18 patients in the late stages of Parkinson’s, a remarkable 85.77% presented with nearly zero complications following the surgery alongside a successful reduction in symptoms including bradykinesia, posture gait, and stability of axial skeleton. These improvements potentially allow patients to extend their life expectancy by up to 8 years^32^. Since its introduction, DBS surgery has been utilized in Pakistan for treating dystonia, one of the motor consequences of stroke^33^. Additionally, the procedure might be modified for improving mobility and post-stroke rehabilitation, particularly in light of findings from the EDEN trial supporting its use for recovery of upper limb function following a stroke^19^. Therefore, DBS could be a major revolution in the management of stroke and post-stroke-related motor disorders in the healthcare context of Pakistan.

DBS is an extensive process that requires prolonged periods of pre and postoperative care, along with a 10-month follow-up once the experimental treatment phase concludes^19^. Sustaining this regimen in Pakistan, where the rate of follow-up after initial consultation is remarkably low, poses a significant challenge which can possibly result in complications and failure of the program. There are currently only five facilities nationwide that are equipped with the necessary technological infrastructure needed to provide DBS surgery, all situated in urban areas^31^. Furthermore, a comprehensive DBS team of specialists such as a movement disorder neurologist, neurosurgeon, speech therapist, physical and occasional therapist, and psychologist is essential for patient selection for DBS surgery^31^. Assembling such a team would be a formidable challenge in Pakistan, where there is a critical shortage of trained healthcare professionals—with only one neurologist for every million people^10^.

Several procedures are also performed as part of the pre-operative workup to identify any contraindications; these include brain MRI to rule out structural abnormalities, and verbal fluency testing before initiating surgery, as DBS has been known to negatively impact speech^31^. The DBS surgery alone would prove to be costly, even before factoring in the cost of pre and postoperative care. A 2020 study estimates the cost of DBS surgery for Parkinson’s disease in Pakistan ranging between two and three million PKR^31^. The exact cost of DBS surgery for stroke in Pakistan is unknown, but based on previously published data, it is expected to impose a significant financial strain.

However, DBS has the potential to significantly transform post-stroke rehabilitation in Pakistan. In contrast to other procedures such as tPA, which must be administered within 4.5 hours of the onset of symptoms^1^, DBS surgery can be undertaken days or even months following a stroke attack^20^. This time difference is monumental in a country like Pakistan, where patients typically only seek care in emergencies, and initial symptoms are frequently disregarded. Given the scarcity of trained neurologists in the country, Pakistani-US MD neurologists have teamed up with the Pakistan Society of Neurology (PSN) to spread awareness of DBS among Pakistani neurologists through workshops, conferences, and even mini fellowships^31^. The Pakistan Stroke Society is also working to establish more stroke centres in various rural and urban areas of the country. Eighteen of the 40 neurology centres in the country were approved for training by the Fellowship of College of Physicians and Surgeons (FCPS) in 2017^8^. These developments illustrate the ongoing efforts of various organizations in Pakistan to improve stroke care and bring it up to international standards, as well as the potential of DBS for post-stroke rehabilitation in Pakistan.

## Long-term efficacy of DBS for Pakistan

In Pakistan and other LMICs in South Asia, significant differences exist in the epidemiology of stroke when compared with the Western countries. These differences include a higher occurrence of stroke at younger ages, a higher prevalence of haemorrhagic stroke, and higher age-specific prevalence rates among women^34^. The role of a stroke caregiver is typically served by a close family member, as there are no inpatient rehabilitation services or structured chronic home support services^35^. A study evaluating the quality of life after stroke in Pakistan reported that stroke survivors were young, mostly depressed, and their quality of life was significantly affected by functional dependence, depression, and neurologic pain. On the other hand, the caregivers were also young, with over half of them being female. The results from the study revealed that stroke caregivers in Pakistan commonly faced frustration, isolation, physical burden, and severe financial crises. There was also an increased incidence of altered eating patterns, type II diabetes mellitus and hypertension among the caregivers^6^.

When discussing the long-term efficacy of DBS for Pakistan, it is crucial to recognize the massive scale of stroke incidence in this populous country, alongside the lack of institutionalized support for chronic disabilities. The reported lifetime prevalence of stroke symptoms in Pakistan is about 19%^36^, yet there is not a single comprehensive inpatient rehabilitation unit available^37^. The introduction of the novel DBS procedure holds promise for restoring motor function, regardless of the time elapsed since the stroke episode.

In the longer run, the widespread application of DBS, serving a substantial population of stroke survivors could potentially reduce dependency on caregivers, marking a significant milestone by alleviating physical and mental health burdens in both the groups. This would additionally benefit the society, as both stroke survivors and caregivers are relatively younger individuals who can contribute actively to economic productivity and social wellbeing.

## Limitations of our review

It is imperative to address the limitations of our review to pave the way for further research and innovations. Firstly, we have only included studies published in the English language, potentially overlooking relevant studies on this topic published in other languages. We only included publicly available data from studies conducted up to April 2024, which may pose a limitation on the time sensitivity of this review as more extensive trials and studies on this topic may be conducted in the future. Furthermore, our study does not provide a comprehensive review of the cost-effectiveness of DBS in comparison to other treatment modalities for post-stroke rehabilitation in Pakistan, which is critical for healthcare decision-making. The effect of DBS on particular patient categories, such as the elderly, or those with uncontrolled diabetes or hypertension was not discussed. Another limitation of our analysis is the heavy reliance on the EDEN trial, conducted in Ohio, USA, which features demographic characteristics substantially different from Pakistan. The trial did not include patients younger than 40 years of age, which is a significant limitation given the high prevalence of stroke in the younger population in Pakistan.

Given the rapid advancement of research, it is critical to acknowledge the possibility of new results that may influence the conclusions presented in this study. Although our research provides significant insights, it is critical to review its findings in light of the limitations noted.

## Conclusion

The objective of this review was to critically evaluate the efficacy of DBS for addressing post-stroke complications, highlighting insights from the recent EDEN trial and considering its scalability in Pakistan, a country with a notably high incidence of stroke. DBS has proved to be successful in Pakistan in very little time. Since its inception in 2014, DBS surgery has been successfully performed on over 100 Parkinson’s patients at the Lahore General Hospital^33^. The individualized nature of DBS surgery means different frequencies of electrical stimuli can be given per the severity of the patient’s symptoms^18^. This opens a pathway for DBS to be adapted for various neurological disorders, including stroke, in Pakistan and worldwide. However, before DBS is adapted for post-stroke rehabilitation in Pakistan, its shortcomings—such as its limited availability, high cost and lack of clinical trials among participants of South Asian descent—must be addressed. Nevertheless, DBS is a technology worth looking into more because of its potential for wider medical applications, which might completely change the way that many movement disorders are treated. Researchers, clinicians, and policymakers in Pakistan must work together to advance this novel therapy to fully realize its benefits and enhance the lives of people with stroke-related disorders. More research is therefore needed to fully tap the potential of DBS for broader clinical applications.

## Ethical approval

This article does not contain any studies with human participants or animals performed by any of the authors.

## Consent

Consent was not required as no human participants were involved in this study.

## Source of funding

This review did not receive any funding from agencies in the public or private sector.

## Author contribution

M.I.: writing of the work. A.A.: writing, reviewing and editing of the work. N.A.K.: writing of the work. U.K.: writing of the work. A.S.: writing of the work. A.A.: writing of the work.

## Conflicts of interest disclosure

The authors declare no conflicts of interest.

## Research registration unique identifying number (UIN)

This study did not involve human subjects.

## Guarantor

Ayesha Azhar.

## Data availability statement

Data sharing is not applicable to this article.

## Provenance and peer review

Not applicable.
